# Peripheral parenchymal organ volume plays a role in neurodegenerative diseases: A Mendelian Randomization Study

**DOI:** 10.1002/alz.094723

**Published:** 2025-01-09

**Authors:** Dinghao An, Yun Xu

**Affiliations:** ^1^ Peking Union Medical College, Beijing China; ^2^ Affiliated Drum Tower Hospital of Medical School, Nanjing University, NANJING, Jiangsu China

## Abstract

**Background:**

Some studies have reported that neurodegenerative diseases can have different effects on the structure and function of peripheral organs, including organ volumes, and we wondered whether there is a genetic causal relationship between the two and whether changes in the volumes of parenchymal organs can affect the risk of developing neurodegenerative diseases.

**Method:**

Our SNP data were obtained from several different databases, including UKB and IEU OpenGWAS, and we analyzed them using bidirectional Mendelian randomization followed by comprehensive robustness analysis.

**Result:**

For traditional genetic threshold: belly subcutaneous fat tissue volume can increase the risk of Alzheimer’s disease. Spleen volume can decrease the risk of amyotrophic lateral sclerosis. Pancreas volume may decrease the risk of amyotrophic lateral sclerosis. Dementia with Lewy bodies can decrease the risk of visceral fat tissue volume change while increasing the risk of spleen volume change. Alzheimer’s disease can decrease the risk of lung volume change, liver volume change, visceral fat tissue volume change, and pancreas volume change while increasing the risk of spleen volume change. For expanded genetic threshold: lung volume change can increase the risk of amyotrophic lateral sclerosis. Alzheimer’s disease can decrease the risk of belly subcutaneous fat tissue volume change, and visceral fat tissue volume change, and increase the risk of lung volume change.

**Conclusion:**

We found that some neurodegenerative diseases affect parenchymal organ volumes and that changes in the volumes of some organs increase the risk of developing neurodegenerative diseases, particularly the volumes of lung, spleen, pancreas, viscera fat tissue, and belly subcutaneous fat tissue. This may provide some new evidence and insights into the pathogenesis and comprehensive management of neurodegenerative diseases.

